# Analysis of Vaginal Microbicide Film Hydration Kinetics by Quantitative Imaging Refractometry

**DOI:** 10.1371/journal.pone.0095005

**Published:** 2014-04-15

**Authors:** Matthew Rinehart, Sheila Grab, Lisa Rohan, David Katz, Adam Wax

**Affiliations:** 1 Department of Biomedical Engineering, Duke University, Durham, North Carolina, United States of America; 2 University of Pittsburgh, School of Pharmacy, Magee Womens Research Institute, Pittsburgh, Pennsylvania, United States of America; 3 Department of Obstetrics and Gynecology, Duke University, Durham, North Carolina, United States of America; Brandeis University, United States of America

## Abstract

We have developed a quantitative imaging refractometry technique, based on holographic phase microscopy, as a tool for investigating microscopic structural changes in water-soluble polymeric materials. Here we apply the approach to analyze the structural degradation of vaginal topical microbicide films due to water uptake. We implemented transmission imaging of 1-mm diameter film samples loaded into a flow chamber with a 1.5×2 mm field of view. After water was flooded into the chamber, interference images were captured and analyzed to obtain high resolution maps of the local refractive index and subsequently the volume fraction and mass density of film material at each spatial location. Here, we compare the hydration dynamics of a panel of films with varying thicknesses and polymer compositions, demonstrating that quantitative imaging refractometry can be an effective tool for evaluating and characterizing the performance of candidate microbicide film designs for anti-HIV drug delivery.

## Introduction

Thin polymeric films have been utilized as dosage forms for oral and topical delivery of drugs. Vaginal films have been commercially available as contraceptive products for many years [1]–[4]. Now, vaginal films are being developed as topical drug delivery vehicles for the prevention of infection and spread of HIV/AIDS [5]–[7]. They have many favorable qualities as drug delivery platforms: films allow localized drug delivery at the most common site of transmission (the vagina), are inexpensive to manufacture, may be applied without an applicator, do not leak, and are highly-stable, portable products that are easily stored for long periods of time without risk of drug degradation [8].

Topical drug delivery by films and other vaginally administered microbicide products (e.g. gels) places requirements not only on the drug release rate but also on subsequent spatial distribution of the active pharmaceutical ingredient (API). This derives from the spreading and hydration behavior of the delivery vehicle [9]–[11]. However, most quantitative methods of evaluation of these products have focused on measuring bulk material physicochemical properties and macroscopic disintegration and dissolution/drug release rates. Film disintegration testing is regularly a part of product development efforts. However, standard USP methods for disintegration testing require testing in large volumes of medium. In order to create a more biorelevant assessment of disintegration, a subjective visual assessment has been used in a lower volume of medium reflective of the small amount of fluids present in the vagina [12], [13]. Although film uptake of fluid and consequent swelling, disintegration, and dissolution, are central to drug delivery by the film, there has been little study of the spatial behavior of film swelling, which underlies the drug release and delivery [12], [13]. As computational film dissolution models are developed [14] and more advanced films are designed with complex dissolution and delivery characteristics, there is a need for objective and quantitative methodology that can verify that film formulations function as intended, delivering APIs to tissue with both the requisite spatial and temporal concentration distributions.

We have previously demonstrated an optical characterization method based on quantitative phase microscopy (QPM) to measure spatial distributions of polymer volume fractions of dissolving polymer films [15]. This technique produces spatial maps of the integral refractive index (RI) throughout a film sample from a single temporal imaging frame. Interferometric phase microscopy has also been used in several cell studies as an effective method for analyzing dynamic changes in water content and cell dry mass [16]–[18]. In the work presented here, we further develop our technique into a quantitative assay and incorporate spatiotemporal analysis of mass transport to present the first comparison of the structural changes of different antiretroviral containing films with varied compositions and geometries.

First, we describe an optimized optical configuration for standardized and comparable experiments. Then, using reference measurements of the refractive index of each sample, we show how to compute the volume fraction and mass density of the film material and water, yielding two-dimensional spatial maps of hydration and structural disintegration. We compare these data for a panel of film candidates loaded with tenofovir (TFV), an antiretroviral drug being studied for vaginal administration for topical pre-exposure prophylaxis. This drug is in the midst of multiple clinical trials, the first of which showed its promise in topical vaginal prophylactic against HIV infection [19]–[21]. Finally, we show that both the polymer composition and film geometry affect not only film hydration rate, but also the spatial distributions of polymer film material.

## Methods

### 2.1 Quantitative Phase Microscopy Setup

**Figure 1:**
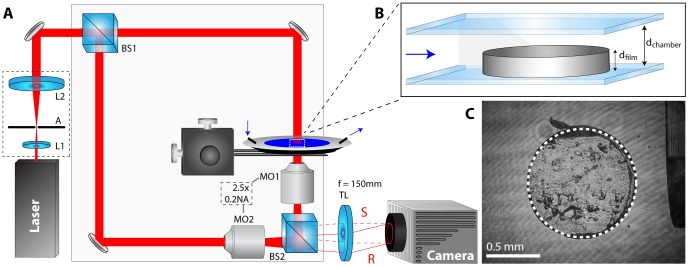
Microscope setup and hydration assay configuration. (A) Experimental schematic of quantitative phase microscope. (B) Film geometry within the chamber at time t = 0. The blue arrow indicates lateral water flow which initiates hydration. (C) Typical interferogram of a 1-mm-diameter film sample with the phase calibration ramp visible in the microscope’s field of view (right edge).

The imaging device used for these experiments is a custom interferometer designed for quantitative phase microscopy, as illustrated in fig. 1A [15]. A beam from a helium-neon laser (λ = 632.8 nm) is spatially-filtered and magnified using lenses L1 and L2 along with aperture A, and is then split into sample and reference arms, S and R, at BS1. The light from the sample arm passes through the sample, which imparts a spatial pattern of phase delays upon this beam. Microscope objective MO1 (2.5× A-plan, Zeiss) is in a 4F imaging configuration with tube lens TL and produces a magnified image of a 2.0 mm×1.5 mm field of view onto the plane of the camera (Flea3.0, Point Grey). The light from the reference arm passes through a matched objective MO2 and recombines with the sample light at beam splitter BS2 before being imaged onto the camera by TL. A lateral shift in the alignment of both the reference beam and MO2 relative to the signal beam path produces a high frequency linear fringe pattern.

### 2.2 Phase Image Processing

After image acquisition, the off-axis interferograms are digitally processed to recover phase images of the relative optical delays associated with the sample [15], [22]. The Fourier transform of this off-axis interference pattern yields distinct autocorrelation and crosscorrelation terms arising from the optical mixing of the reference and sample light fields. One of the cross correlation terms is first isolated by spatial filtering, then re-centered in frequency space, and is finally inverse Fourier transformed to recover the complex wave information [22]. Taking the phase of this complex-valued data produces a two-dimensional map of the relative phase difference between the reference beam and the sample beam. The recovered phase delays are mathematically limited in range to ±π, with longer or shorter delays being ambiguously measured modulo-2π. In order to recover a true representation of the relative phase delays across the field of view, spatial [23] and temporal [24] unwrapping algorithms are combined as described previously [15]. Under the assumption that the true phase distribution does not change by more than π/2 between two adjacent data points in either time or space, these algorithms produce unambiguous phase images.

The recovered quantitative phase measurements are further processed to produce film polymer volume fraction and hydration maps. First, a phase image of the flow chamber filled with water is subtracted from each image to remove system phase artifacts and yield the relative phase delays induced by only the film material, 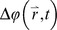. Then, using the chamber height as measured with digital calipers (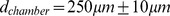), the average refractive index, 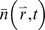, is calculated as:



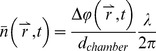
(1)


It is important to note that 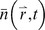 is the average RI over the entire axial thickness of the chamber sample, and therefore represents a linear combination of the component refractive indices weighted by their relative heights as given in eqn. 2:
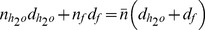
(2)


The height of the sample chamber along with the xy-lateral dimensions of the microscope form a voxel whose RI is a linear combination of the RIs of the water, 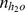, and film, 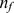, components weighted by the heights of the water and film, 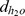 and 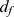, respectively. Upon decomposition of the measured RI values into percent-volumes of film polymer and water, quantitative maps are produced. From these, the percent volume of film, *F*, (i.e. volume fraction of polymer) at each spatial point is calculated:



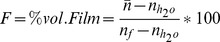



The quantitative phase microscope and processing methods described above achieve a temporal phase stability of 13.5 mrad, corresponding to a refractive index sensitivity of 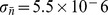 [15]. This measurement sensitivity along with the measurements and uncertainties of 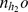


The quantitative phase microscope and processing methods described above achieve a temporal phase stability of 13.5 mrad, corresponding to a refractive index sensitivity of 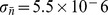 [15]. This measurement sensitivity along with the measurements and uncertainties of 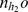 and each film’s 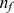 (Table 1) are propagated through eqns. 3 and 4 to yield a film fraction measurement precision ranging from 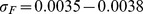 percentage points (pp).

**Table 1:** Refractive indices & measured dissolution parameters of specific film formulations.

	dn/dc (ml/ml)	Density, 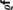 (g/ml)	α (ml/g)	Refractive Index	τ_H_ (m:ss)	Λ	Γ
**H_2_O**		1.000		1.3329±.0001			
**T1A**	0.154±.002	1.183	0.130±.002	1.486±.002	5∶16	.076±.002	0.165±.033
**T2A**	0.144±.001	1.118	0.129±.001	1.477±.001	5∶44	.105±.003	0.253±.100
**T3A**	0.151±.008	1.315	0.115±.006	1.485±.008	8∶52	.016±.001	0.061±.031
**T1B**	0.154±.002	1.180	0.130±.002	1.486±.002	7∶48	.116±.003	0.125±.007

α values are given as mean ±95% CI. τ_H_ is hydration time; Λ and Γ given as mean ± standard deviation.

### 2.3 Microbicidal Film Characterization

Microbicidal films were manufactured in sheets that are approximately 8 inches × 12 inches using a solvent casting method similar to that described by Akil, et al [12]. Films were cast onto substrate and dried before being cut into 1 inch by 2 inch individual film strips using a die press. Individual film strips were analyzed for drug content using high pressure liquid chromatography methods and were found to contain approximately 20 mg of tenofovir. Water content was measured using a Karl Fisher titration apparatus. Film thickness was measured with mechanical calipers with a measurement resolution of ±10 µm. Finally, the RIs of each distinct film composition, as well as that for the deionized water used for the flow experiments, were measured separately using a bulk refractometer (Bellingham & Stanley, RFM 340). Since this is a surface measurement device, equilibrium serial dilutions of the polymer film samples with water were prepared. Fitting the RI measurements of each film’s dilution series to a linear model as 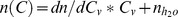 where *C_v_* is the volume concentration, yielded a value of 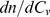 for each film. Each film’s density was then calculated according to the compositions listed in Table 2 and used to convert 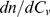 to RI increments, *α*, which is a parameter that is widely used in interferometric mass analysis of biomaterials [18], [25], [26]. We evaluated four distinct films (Table 2): T1A, T2A, and T3A, which are tenofovir-loaded films, 120 µm thick, which differed in polymer composition, and T1B, which has the same composition as T1A but is twice as thick.

**Table 2:** Composition and thicknesses of films analyzed in the study.

	Composition prior to evaporation									Post-evaporation properties		
	PVP	MC	HPMC	HEC	NaCMC	TFV	GLYC	NaOH	H_2_O	H_2_O	Thickness (µm)	RI
**T1A**	–	–	6	6	2	2	2	0.28	81.72	5.47	120	1.486
**T2A**	–	–	7	7	–	2	2	0.28	81.72	7.15	120	1.477
**T3A**	10	2	–	–	2	2	3.6	0.28	80.12	10.51	120	1.485
**T1B**	–	–	6	6	2	2	2	0.28	81.72	7.31	240	1.486

All values are given as % w/w, except for thicknesses and RIs.

Ingredient abbreviations: hydroxyethyl cellulose (HEC), hydroxypropyl methylcellulose (HPMC), carboxymethylcellulose sodium (NaCMC), Tenofovir (TFV), glycerin (GLYC), sodium hydroxide (NaOH), polyvinylpyrrolidone K90 (PVP), methyl cellulose (MC), water (H_2_O).

### 2.4 Film Hydration Assay

Samples for the hydration assay were cut from the film strip using a 1 mm-diameter biopsy punch and then placed on a round coverslip in close proximity to a reference structure. This structure is a transparent physical ramp (Sylgard 184, Dow Corning) that is epoxied to the coverslip in order to provide a constant refractive index reference in the field of view during the experimental time course [15]. Gentle pressure was applied to the film with a blunt pair of tweezers in order to partially attach the sample to the coverslip. This ensured that the film remained centered in the field of view when water was added at the beginning of the experiment.

After placement of a film sample, a coverslip was inserted as the top of a lateral flow chamber (Bioptechs, FCS3 chamber). A 250 µm thick gasket sealed the chamber and thus allowed both top and bottom coverslips to make contact with the calibration ramp structure. The sample was centered in the QPM field of view, and then water was injected with a hand syringe into the chamber from an inlet port until it completely immersed the sample and flowed to the outlet port. As soon as the water reached the outlet port (∼2–3 seconds after film immersion), flow was halted. Images were acquired at 2 s intervals over a time course of 20 minutes as the film dissolved. After a defined 20 minute hydration period, a large volume of water was flushed through the chamber to clear all film material from the field of view. An interferogram was then captured with the water-filled chamber as a reference phase measurement. Each interference image was then processed as described in section 2.1 to yield film volume fraction data (approx. 2 s per image, 20 min total processing time per dataset), which became an output measure of hydration for comparison across repeated experiments and varied film compositions. The hydration assay was repeated three times for each film type.

### 2.5 Comparative Analysis of Films

Film volume fraction images were calculated from the QPM measurements as described in section 2.2. Although films T1A–T3A have unique compositions and densities, their initial geometries are identical and therefore their volume fractions are directly comparable during hydration and dissolution. Representative images of film volume fraction were selected for direct comparison at specific time points. The volume fraction of film material remaining within the initial area was also compared by manually selecting the center location of each sample and averaging the volume fraction remaining within a 1 mm-diameter area (fig. 1C).

**Figure 2:**
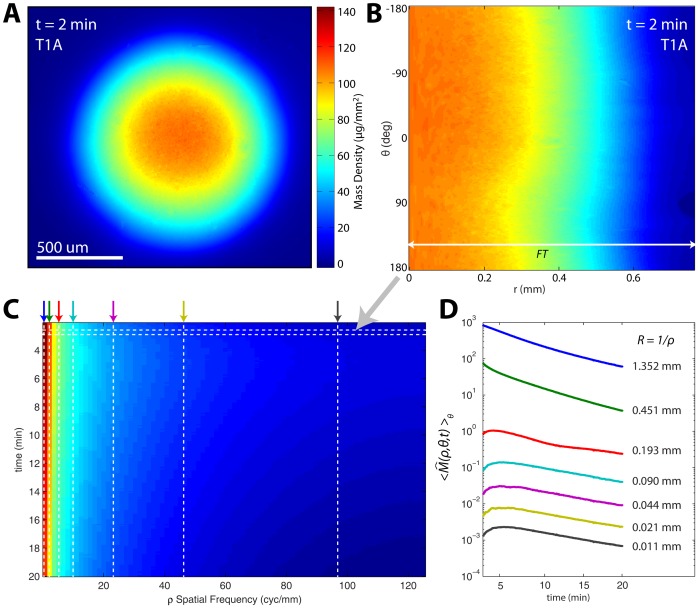
Spatial Fourier analysis of mass density: (A) Mass density image of film T1A at t = 2 min; (B) Polar-coordinate representation of data in (A). Mass density distributions are then Fourier-transformed in the radial direction and averaged azimuthally to produce (C) power spectral density over time. (D) As the film hydrates and dissolves, each spatial frequency component decays with different temporal dynamics.

Additionally, differences in hydration dynamics were analyzed by quantifying spatial distributions of the polymer dry mass. The mass density, which indicates polymer concentration, was calculated from volume fraction data using the known geometry of the flow chamber and the density of each film (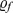 in g/ml, see Table 1):
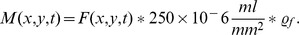



Mass density images were interpolated from Cartesian (x, y) to polar (r, *θ*) coordinates, (Figs. 2A and B), and axiosymmetric radial profiles were calculated by taking the median value over *θ* as a robust measure of central tendency.

Hydration dynamics of the varied polymer films were further compared using spatial Fourier analysis (SFA) of the mass density distributions. Polar mass density distributions (Fig. 2B) were Fourier transformed in r, and averaged over θ to produce 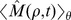, which is a measure of how each radial spatial frequency evolves over time (Figs.2B and C). The dynamics of each film’s hydration process were then compared by examining the temporal dynamics of specific spectral components (Fig. 2D).

## Results

### 3.1 Film Characteristics Summary

Table 2 summarizes the initial compositions, the residual water content after evaporation, thickness, and refractive indexes of the film compositions analyzed in this study. Films T1A and T2A are more chemically similar to each other than T3A, containing only a 2% difference in fractional composition. Meanwhile, T3A substitutes two unique polymers and contains almost double the content of glycerin relative to T1A and T2A. Notably, films T1A and T3A contain NaCMC (n = 1.515) and have higher refractive indices than T2A, which does not contain NaCMC.

### 3.2 Refractive Index Measurements of Water and Film Samples

Table 1 summarizes the RI increments (see section 2.3) and extrapolated RI values of the water and three distinct film polymer compositions used in the hydration experiments. The error bars correspond to the 95% confidence bounds of the fit model. Films T1A, T2A and T1B all exhibit similar RI increments, α, while T3A was found to have a significantly lower refractive index increment.

### 3.3 Effects of Film Thickness on Hydration Behavior

**Figure 3:**
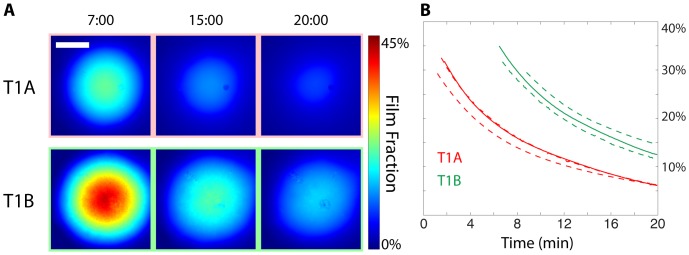
Comparison of two films with different thicknesses, T1A (120 µm) and T1B (240 µm). (A) Calculated film fraction at 7, 15, and 20 minutes. (B) Film fraction remaining within the initial circular film area during hydration. The three repeats of T1A (red) show a distinctly faster hydration rate than the repeated measurements of samples from the T1B film (blue). Solid lines indicate experimental data depicted in (A), while dashed lines are data from repeated experiments with identical conditions. Scale bar: 0.5 mm.

Figure 3 compares representative film volume fraction images (fig. 3A) and summary (volume average over the entire film, r = 0 mm to r = 0.5 mm) volume fraction time curves (fig. 3B) for two film types with identical polymer compositions but different thicknesses, T1A and T1B. The hydration data contains several notable features. First, T1A is half as thick as T1B and thus contains half the volume of polymer material as T1B initially. Additionally, the volume of T1A polymer within the initial area is about half that of T1B at each time point because both films spread at approximately the same rate. Finally, the thicker film, T1B, exhibited phase wrapping artifacts for the initial 7 minutes of the experimental time course, whereas the phase data from the thinner film T1A were free of wrapping artifacts within 2 minutes of initial hydration. The consequence of this behavior difference is discussed below.

### 3.4 Film Hydration Comparison: Varying Polymer Composition

**Figure 4:**
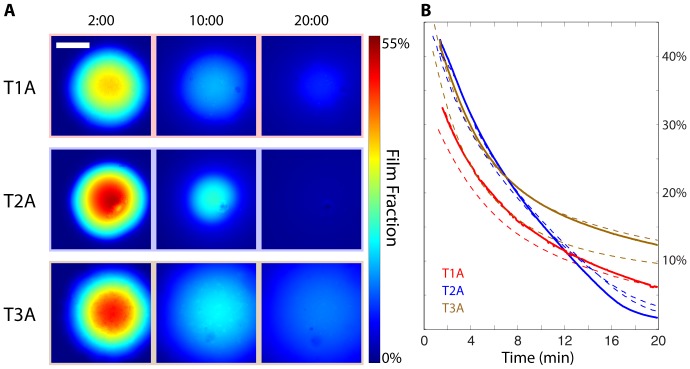
Comparison of three distinct film formulations as summarized in Table 2. (A) Calculated film fraction at 2, 10, and 20 minutes. (B) Film fraction remaining within the initial circular film area during hydration; Solid line corresponds to the hydration time course represented in (A), dashed lines correspond to triplicate repeat experiments and demonstrate assay variability/repeatability.

The hydration experiments were repeated with the three films of different compositions, to explore the effect of composition on hydration dynamics (fig. 4). Here, T1A and T3A appear to have similar temporal hydration profiles but exhibit early kinetic differences, along with a potential variation in initial thickness of ±10 µm. These result in different film volume fractions after the first two minutes of the experimental time course. While T2A appears to contain the largest volume fraction of film material at its center two minutes after hydration, this central concentration diminishes rapidly and contains the least fraction of film material after 20 minutes.

### 3.5 Azimuthally-averaged radial profiles

**Figure 5:**
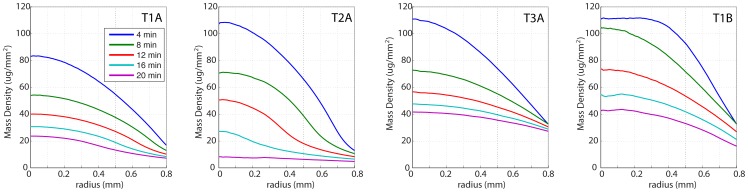
Azimuthally-averaged radial mass density profiles.

The effects of varied polymer compositions and thickness are further examined in concentration profiles shown in fig. 5. Azimuthal averaging has removed small-scale features and allows examination of the macroscopic radial shapes exhibited by the polymers during dissolution. T1A and T2A exhibit a pronounced central core: after initial hydration, the films retain an inflection point within the field of view that migrates inward during further dissolution. T3A differs significantly in composition (see Table 2), and lacks this inflection feature. Interestingly, the thicker film T1B, which is compositionally identical to T1A, lacks this feature as well. Because T1A’s inflection point is near the edge of the field of view, we expect that the same feature is not seen in T1B’s mass density profiles both because it occurs beyond the field of view and also because the inward migration of the inflection point is expected to occur later than the final time point of this experiment.

### 3.6 Spatial Frequency Analysis

**Figure 6:**
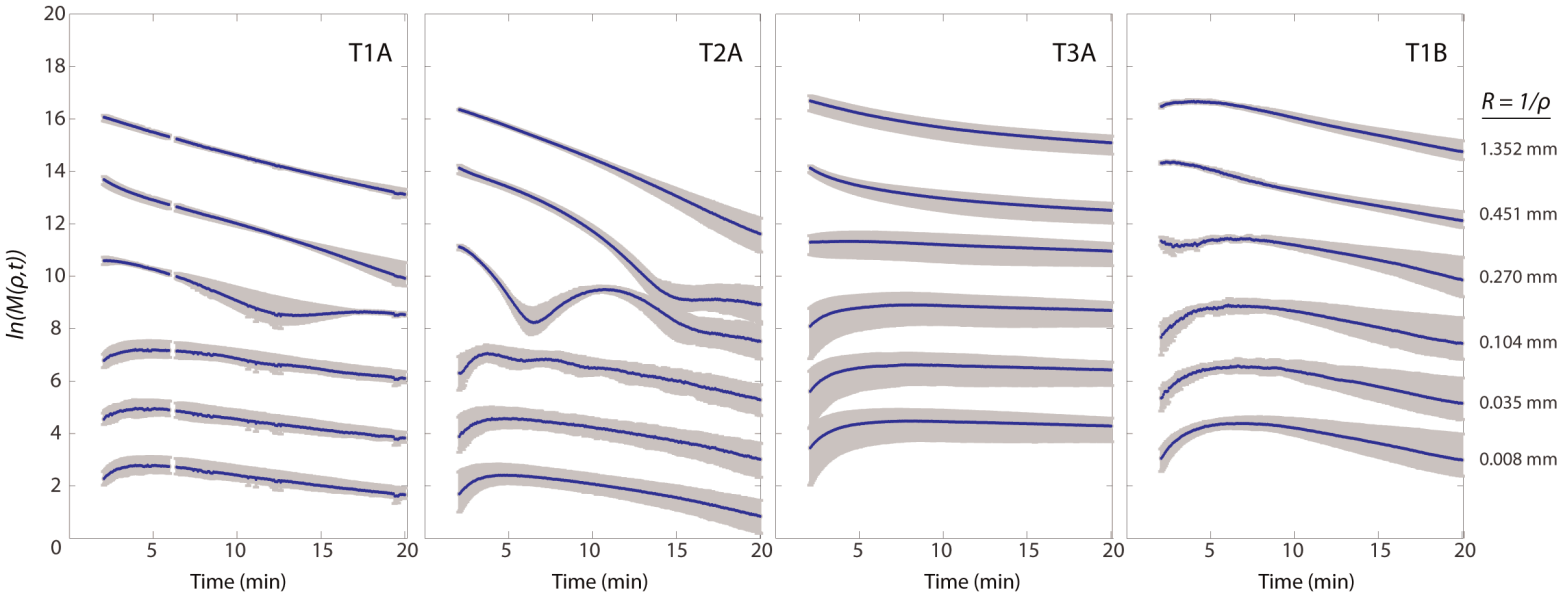
Spatial frequency time dynamics. Selected characteristic length scales demonstrate early hydration and subsequent dissolution. Blue lines and error bars correspond to the mean and standard deviation of three repeats of each film type.

Spatial frequency analysis indicates distinct behavior at characteristic length-scales given by R = 1/ρ. Relative increases and decreases in the amplitude of specific length scales occur as dissolution proceeds by mechanisms such as polymer chain disentanglement, fragmentation, swelling, and diffusion [27]–[29]. Pure diffusion, as governed by Fick’s second law, predicts that the power in each spatial frequency component will decay as:



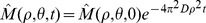
where 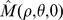 is the initial mass density distribution and D is the diffusion coefficient [30], [31]. Figure 6 illustrates that diffusion does not appear to be the dominant mechanism of mass transport for any of the compositions or thicknesses, although each film’s hydration behavior differs significantly. For all films, amplitudes for characteristic length scales less than 0.27 mm increase in relative contribution during the first several minutes of hydration, which we attribute to swelling and polymer chain disentanglement. As dissolution continues, all curves approach uniform decay rates. Films T1A and T2A also exhibit damped oscillations in length scales 0.2 mm<R<0.5 mm due to complex polymer-solvent interactions. The smallest length scales correspond to the resolution limit of the interferometric imaging system.

Each spatial frequency time series calculated by SFA is divided into two regimes: (1) an initial hydration regime and (2) a dissolution regime. These two regimes are divided at τ_H_, the time where all spatial frequencies have passed their maximum value. The hydration times for films T1A and T2A, which have similar polymer compositions, are 5∶16 and 5∶44, respectively (times represented as m:ss). T3A has a much longer τ_H_ of 8∶52. Doubling the film thickness while keeping the polymeric composition identical (T1A = 120 µm, T1B = 240 µm) increases τ_H_ from 5∶16 to 7∶48.

**Figure 7:**
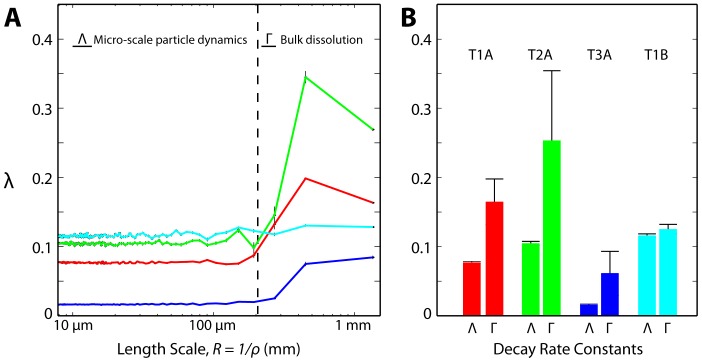
Spatial frequency decay rates. (A) Each characteristic length scale is fit over the time interval of (τ_H_,20∶00) to an exponential decay, 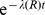. 95% confidence intervals of the fits are shown as black bars. (B) Average bulk decay rates (Γ) and micron-scale particle decay rates (Λ) of each sample. Black bars show standard deviation (See Table 1 for summary).

Once in the dissolution regime, each characteristic length scale amplitude curve is fit to an exponential decay of the form: 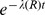 to determine the decay rate, λ. There is a clear difference in decay behavior above and below R = 200 µm (see fig. 7A), where long length scales indicate bulk dissolution dynamics and the small length scales indicate the micron-scale particle behavior of the film. Fig. 7B shows the average decay rates of each film sample’s bulk (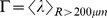) and micron-scale (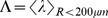) features. While T1A–T3A all have the same physical geometry, T3A exhibits both a lower bulk decay rate as well as slower dissolution of micron-sized particles. All three of these films have larger decay rates at long length scales and approximately constant decay rates at length scales smaller than 200 µm; In contrast to the 120 µm-thick films, T1B’s Γ and Λ decay rates are similar. The variation in decay rates Γ and Λ indicates that polymer composition and geometry affect not only the bulk rate of dissolution, but also the rate at which smaller-sized objects are generated and decay. Each parameter is expected to have a distinct influence on the film’s ability to spread across and deliver API to a target tissue.

## Discussion and Conclusion

In this study, we have demonstrated that quantitative imaging refractometry can be an objective, quantitative tool for investigating differences in the water uptake, hydration, and dissolution behaviors of microbicide films with varying compositions and thicknesses. These processes impact drug release profiles and ultimately underlie drug pharmacokinetics. We have introduced the methodology and illustrated its use for a set of prototype films being evaluated for vaginal delivery of the topical anti-HIV microbicide Tenofovir. To date, no method exists to study the initial stages of film hydration, which are critical to drug release. Thus, the methodology presented here represents a novel technique for studying the spatial distributions of hydration parameters *in vitro*, which can aid in film formulation development and understanding of *in vivo* film behavior.

In the experiments presented here, we compared film prototypes with different thicknesses as well as polymeric compositions. Refractive index measurements of the films enabled calculation of film volume fractions (local, average), mass density profiles, and spatial Fourier analysis that can be used in comparing various film designs. Film T1A (*d* = 120 µm) dissolved consistently quicker than the 240 µm-thick film of the same formulation, and contained approximately half as much material in the initial film area after 20 minutes of hydration (T1A = 6.2%, T1B = 12.5%). Variations in polymer formulation produced films of identical thickness that dissolved at different rates, as demonstrated by the fitted Λ and Γ values. Notably, results here showed that composition and geometry can be altered to produce not only different hydration rates, but distinct bulk- and micro-scale particle dissolution rates that can vary independently. These results illustrate that our new methodology will provide a reference for future modeling and analysis that seek to understand how polymer-solvent kinetics (including polymer chain disentanglement, material cracking, and formation of a glassy transition layer) govern film vehicle spreading as well as microbicidal drug release and delivery to tissues of interest.

The precision, accuracy, and fidelity of this method are bounded by several factors. Each 2π wrapping artifact contributes an error of 2.5×10^−3^ refractive index units, which corresponds to a film volume fraction error of 1.65–1.76 pp depending on the specific refractive index of the film. In comparison, the spatiotemporal phase fluctuations, as limited by the inherent noise of the interferometer, yield a refractive index sensitivity of *σ = *5.5×10^−6^ [15], corresponding to film fraction measurement precision of 0.0035–0.0038 pp and a mass density measurement precision of 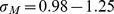 *µg/mm^2^*. The accuracy of the measured film fractions is bounded by the error of the refractive index measurements given in Table 1. While the absolute differences across the four film samples contain some uncertainty due to these calibration measurements, the system precision suggests that the differences in hydration kinetics between specific film repeat measurements (figs.3–7) are deterministically attributable to sample geometric and kinetic variability. It is important to note that the measured phase data contain large errors due to wrapping artifacts during the initial minutes following hydration, rendering data at these early times unsuitable for analysis. After a short period of hydration, the phase images become smooth and easily unwrapped, and thus produce high quality data for the remainder of each experiment.

In conclusion, we have presented and applied a novel methodology for the first objective, quantitative imaging comparison of microbicide film polymer hydration kinetics. Our results demonstrate that film geometry and polymer composition each can have a significant effect on not only the time to hydrate, but also on the specific behavior of the spatiotemporal dissolution profiles. These results can be input to mechanistic analyses of film hydration, disintegration, drug dissolution and release [14], helping to improve our understanding of the determinants of those processes. In turn, they can contribute to improved design of films for effective vaginal delivery of anti-HIV microbicides.
